# Uniquely low stable iron isotopic signatures in deep marine sediments caused by Rayleigh distillation

**DOI:** 10.1038/s41598-023-37254-2

**Published:** 2023-06-24

**Authors:** Male Köster, Michael Staubwasser, Anette Meixner, Simone A. Kasemann, Hayley R. Manners, Yuki Morono, Fumio Inagaki, Verena B. Heuer, Sabine Kasten, Susann Henkel

**Affiliations:** 1 Alfred Wegener Institute Helmholtz Centre for Polar and Marine Research, Bremerhaven, Germany; 2 Faculty of Geosciences, University of Bremen, Bremen, Germany; 3 University of Cologne, Cologne, Germany; 4 MARUM – Center for Marine Environmental Sciences, University of Bremen, Bremen, Germany; 5 School of Geography, Earth and Environmental Sciences, University of Plymouth, Plymouth, UK; 6 Kochi Institute for Core Sample Research, Extra-cutting-edge Science and Technology Avant-garde Research (X-star), Japan Agency for Marine-Earth Sciences and Technology (JAMSTEC), Nankoku, Kochi, Japan; 7 Institute for Marine-Earth Exploration and Engineering (MarE3), JAMSTEC, Yokohama, Japan; 8 Department of Earth Sciences, Graduate School of Science, Tohoku University, Sendai, Japan

**Keywords:** Element cycles, Biogeochemistry, Carbon cycle, Marine chemistry, Geochemistry

## Abstract

Dissimilatory iron reduction (DIR) is suggested to be one of the earliest forms of microbial respiration. It plays an important role in the biogeochemical cycling of iron in modern and ancient sediments. Since microbial iron cycling is typically accompanied by iron isotope fractionation, stable iron isotopes are used as tracer for biological activity. Here we present iron isotope data for dissolved and sequentially extracted sedimentary iron pools from deep and hot subseafloor sediments retrieved in the Nankai Trough off Japan. Dissolved iron (Fe(II)_aq_) is isotopically light throughout the ferruginous sediment interval but some samples have exceptionally light isotope values. Such light values have never been reported in natural marine environments and cannot be solely attributed to DIR. We show that the light isotope values are best explained by a Rayleigh distillation model where Fe(II)_aq_ is continuously removed from the pore water by adsorption onto iron (oxyhydr)oxide surfaces. While the microbially mediated Fe(II)_aq_ release has ceased due to an increase in temperature beyond the threshold of mesophilic microorganisms, the abiotic adsorptive Fe(II)_aq_ removal continued, leading to uniquely light isotope values. These findings have important implications for the interpretation of dissolved iron isotope data especially in deep subseafloor sediments.

## Introduction

Iron (Fe), one of the most abundant elements on Earth, is a redox-sensitive element that mainly occurs as ferrous (II) and ferric (III) Fe. Microorganisms acquire energy by reducing or oxidizing Fe between Fe(II) and Fe(III) redox/oxidation states^1^. These reactions are strongly linked to the element cycles of carbon and sulfur, thus imposing an important driver of global biogeochemical cycles. Dissimilatory Fe(III) reduction (DIR) is among the earliest microbial metabolic pathways on Earth, and Fe(III)-reducing microorganisms might be key inhabitants of the deep and hot biosphere^2,3^. The deep biosphere here refers to marine sediments deeper than 5 m below the seafloor (mbsf) and continues for several hundreds to thousands of meters down into the seabed^4^.

Stable iron isotope analyses are widely applied to trace and decipher Fe sources, transport and reaction pathways in marine environments^5–10^. The ratio of the two most abundant Fe isotopes (^54^Fe and ^56^Fe), commonly expressed as δ^56^Fe (‰), can provide valuable insight into biogeochemical Fe cycling, and may be used as a proxy for microbially mediated processes in modern and ancient marine sediments^11–13^. Notable Fe isotope fractionation occurs during redox processes^14–16^. The most pronounced fractionation of up to − 3‰﻿ compared to the average isotopic composition of igneous rocks (δ^56^Fe = 0.09 ± 0.05‰﻿, 1SD ref.^17^) is caused by coupled electron and Fe atom exchange between Fe(II) and Fe(III) at Fe oxide surfaces during DIR^15,16^. Since microbes preferentially consume ^54^Fe over ^56^Fe, the respective dissolved Fe (Fe(II)_aq_) is isotopically light while the residual Fe(III) becomes progressively enriched in isotopically heavy ^56^Fe^5,16,18^. Iron isotopes also fractionate during abiotic processes, including adsorption of Fe(II)_aq_ on mineral surfaces (preferential adsorption of isotopically heavy ^56^Fe)^15,16,19^ or the precipitation of Fe minerals (fractionation depends on whether the reaction is kinetically controlled or in equilibrium)^14,20–22^. While several studies have focused on Fe isotope fractionation during early diagenesis in shallow (< 5 mbsf) sediments^6,18,23^, no isotopic records exist for dissolved Fe in deep subseafloor (> 5 mbsf) sediments so far.

**Figure 1:**
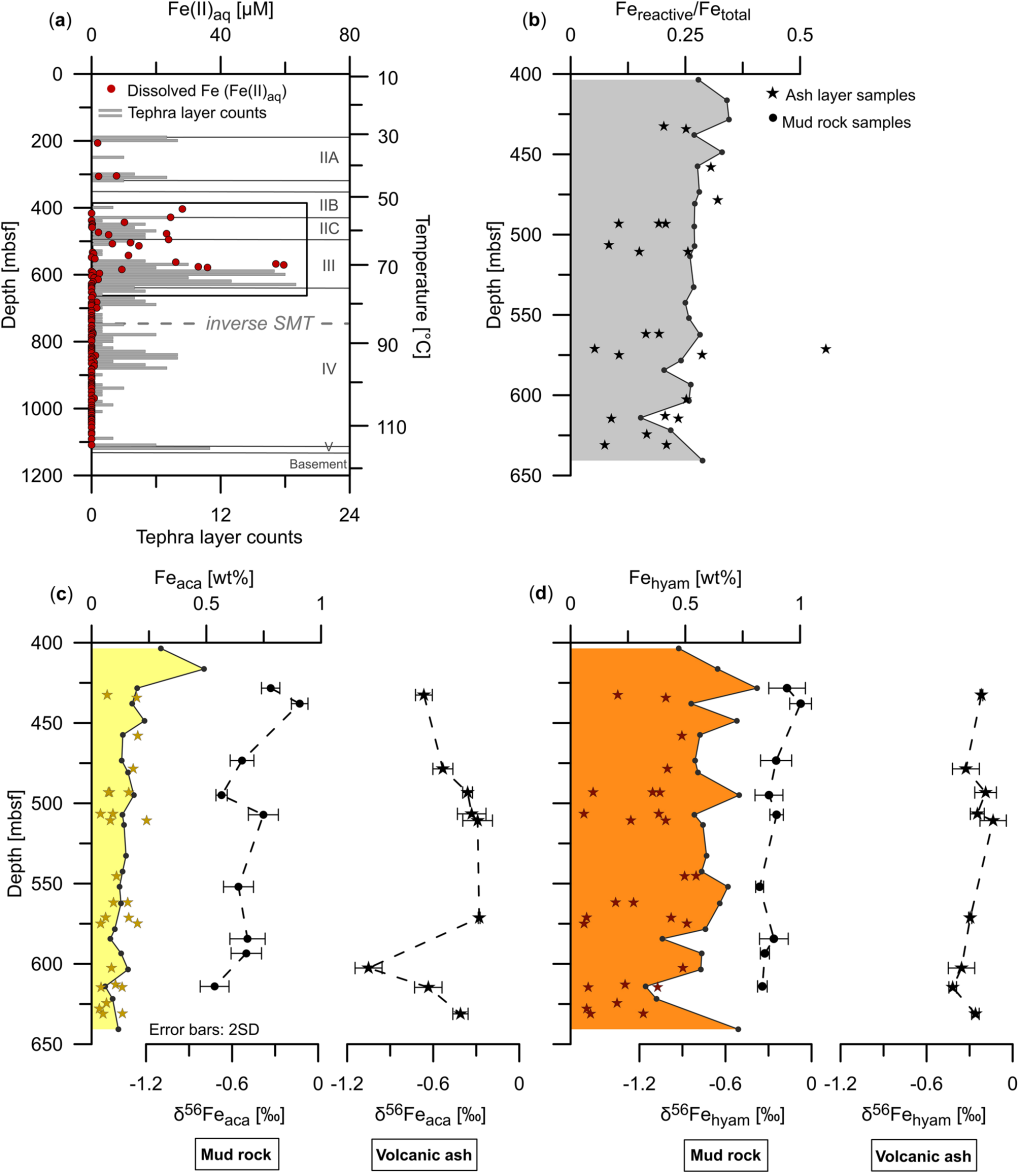
Dissolved Fe (Fe(II)_aq_) concentrations, reactive Fe contents and associated isotopic compositions in mud rock and discrete ash layers at Site C0023. (**a**) Down-core pore-water profile of Fe(II)_aq_ concentrations (red dots) and tephra layer counts (gray bars)^25^. Lithological units (IIA-V; see Supplementary Information) and temperature data are from Heuer et al.^25^ and Heuer et al.^24^, respectively. The gray dashed line shows the location of the inverse sulfate-methane transition (SMT) with a methanic zone above and a sulfate-rich zone below the SMT (see Supplementary Fig. 2 for details). (**b**) Down-core profiles of the reactive Fe (Fe_reactive_: sum of sequentially extracted Fe pools according to Poulton and Canfield^26^) to total Fe (Fe_total_) ratio (Fe_reactive_/Fe_total_). (**c**) Na-acetate-leachable Fe (Fe_aca_) and associated isotopic composition (δ^56^Fe_aca_). (**d**) Hydroxylamine-HCl-leachable Fe (Fe_hyam_) and the associated isotopic composition (δ^56^Fe_hyam_). Error bars indicate the twofold standard deviation (2SD) of duplicate to fourfold measurements. The stars in each panel represent the discrete ash layer samples whereas the dots are the surrounding mud rock samples. Fe_total_, Fe_reactive_, Fe_aca_ and Fe_hyam_ data of mud rock are taken from Köster et al.^31^.

Here, we investigate pore-water and solid-phase samples that were collected during International Ocean Discovery Program (IODP) Expedition 370 from a 1180 m deep hole (Site C0023) drilled in the Nankai Trough off Cape Muroto, Japan. Temperatures of up to 120 °C at the sediment-basement interface and high heat flow characterize Site C0023 (ref.^24^). The aim of the expedition was to explore the temperature limit of microbial life and to identify geochemical and microbial signatures that differentiate the biotic and abiotic realms^25^. Dissolved Fe was detected predominantly in an interval characterized by elevated amounts of volcanic ash layers (Fig. 1a)^25^, suggesting that volcanic ash provides reducible minerals that stimulate microbial Fe reduction and the release of Fe(II)_aq_. To assess the role of ash layers and the availability of Fe phases for biogeochemical processes in the deep and hot biosphere, we performed sequential extractions of reactive Fe phases on discrete volcanic ash and surrounding mud rock samples^18,26^. Since the sediments at Site C0023 are already consolidated^25^, we use the term ‘mud rock’ in the following. By combining δ^56^Fe analyses of pore-water and extracted Fe, another aim was to decipher if the isotopic composition of dissolved and reactive solid-phase Fe is indicative of microbial Fe reduction. We hypothesized that negative δ^56^Fe values in the pore water would be a strong argument for microbially driven processes. However, we find extremely low δ^56^Fe pore-water values that are unlikely to be caused by microbial Fe reduction alone. As the most likely explanation for this finding, we present a Rayleigh distillation model that includes the adsorption of Fe(II)_aq_ onto Fe (oxyhydr)oxide surfaces.

### Differences in Fe contents between ash-bearing layers and surrounding mud rock

In contrast to previous studies in depositional environments that are characterized by abundant ash layers^27–30^, the contents of reactive Fe in ash samples at Site C0023 are significantly lower compared to the surrounding mud rock (Fig. 1b–d). Here, reactive Fe is expressed as the sum of all sequentially extracted Fe pools in relation to total Fe (Fe_reactive_/Fe_total_)^26^.

The Na-acetate step of the leaching sequence predominantly dissolves adsorbed Fe(II) (Fe(II)_sorb_), carbonate-bound Fe and Fe monosulfides (Fe_aca_)^18,26^. The average Fe_aca_ contents in ash layer and mud rock samples are ~ 0.1 wt% (Fig. 1c). Since Fe monosulfides were not detected in the sediments of Site C0023 (ref.^31^), Fe_aca_ represents abundantly present siderite and Fe-rich calcite^25^, whose formation can most likely be attributed to the alteration of volcanic ash^32,33^, and some adsorbed Fe(II). Volcanic ash does not only occur as discrete layers (Supplementary Fig. 3), but also as dispersed ash. Hence, the formation of authigenic carbonate as a result of volcanic ash alteration likely also affected the surrounding mud rock and no significant differences in the Fe_aca_ contents between discrete ash samples and the surrounding mud rock are observed (Fig. 1c).

Hydroxylamine-HCl typically extracts ferrihydrite and lepidocrocite (Fe_hyam_)^18,26^. However, Fe_hyam_ in sediments at Site C0023 was shown to mainly consist of reactive phyllosilicate-bound Fe^31^. It represents the sequentially extracted dominant Fe fraction with contents of up to 1.0 wt% in mud rock^31^ and 0.6 wt% in discrete ash samples (Fig. 1d). The considerably lower Fe_reactive_ and Fe_hyam_ contents in ash-bearing layers indicate that (1) part of the Fe(III) deposited synsedimentary with the tephra has already been used by microbes and is thus not preserved and/or (2) Fe(III) in tephra was originally lower due to the specific chemistry of the volcanic source material. Similar to our findings, reactive Fe contents in ash samples from IODP Site U1229D in the Bering Sea are lower compared to those in the background sediments^34^. The volcanic material at this site is sourced from the Aleutian arc. The lower reactive Fe contents in the ash material are potentially caused by the fact that the Aleutian eruptions are primarily andesitic and rhyoltic in composition^34^. Rhyolitic ash is generally characterized by low Fe contents (~ 2 wt%)^35^. The Ti/Al ratios in the discrete ash samples in our study vary between 0.01 and 0.06 (Supplementary Fig. 4) indicating a dacitic to rhyolitic composition. This is in line with felsic ash derived from the Japanese Islands^36^. Higher Fe contents in mud rock samples are likely due to a high amount of mafic minerals such as pyroxene and amphibole^25^, suggesting that the reactive Fe contents in the discrete ash layers were already lower at the time of deposition. The variability in reactive Fe contents in the discrete ash layers (Fig. 1b–d) might further indicate that the ash layers are from different sources. Nevertheless, we do not rule out the alternative explanation that reactive Fe(III) in the ash layers has already been consumed by microbes and consider a combination of both processes most likely.

### Indications of microbial Fe cycling

**Figure 2:**
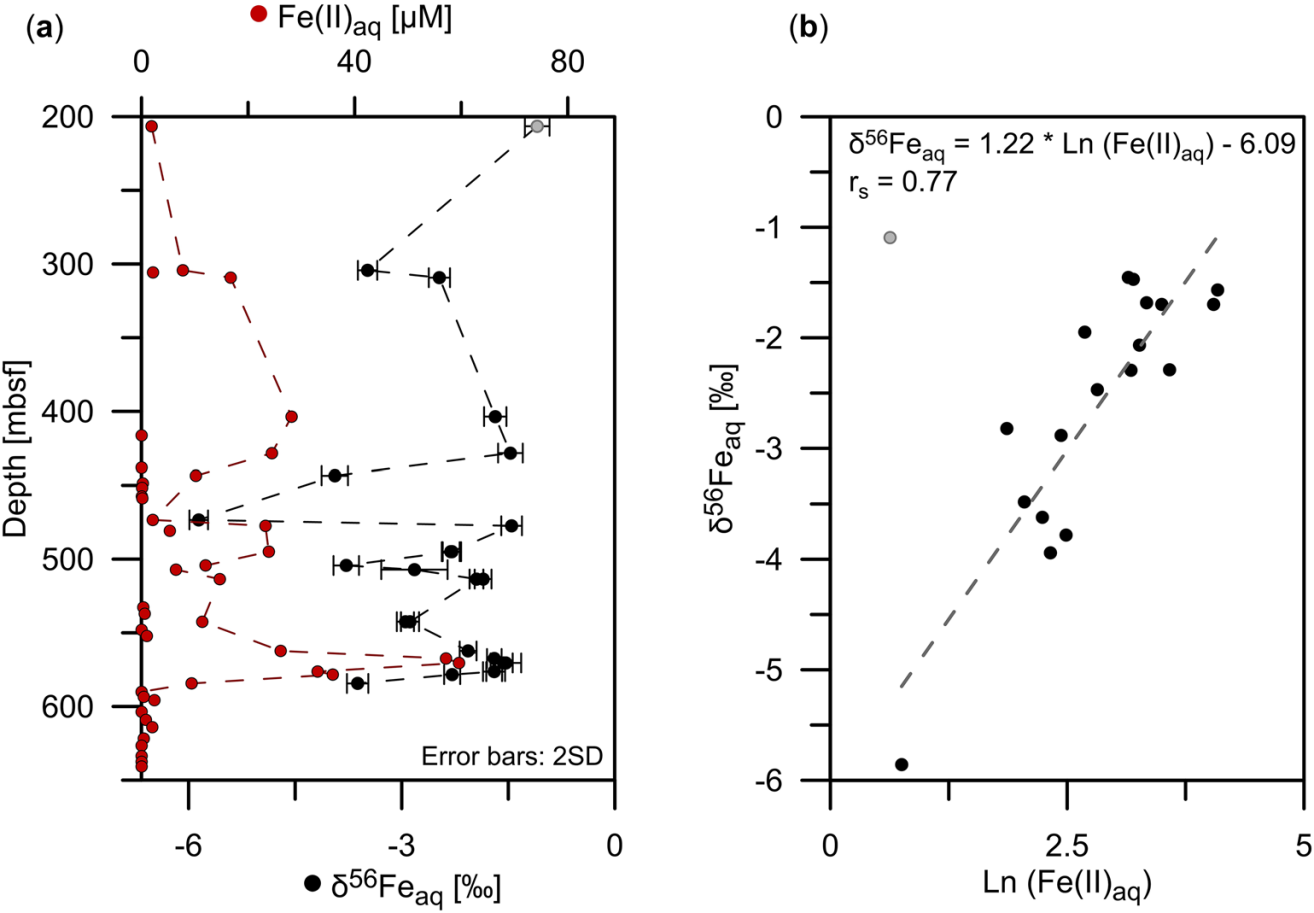
Dissolved Fe (Fe(II)_aq_) concentrations and associated isotopic compositions at Site C0023. (**a**) Close-up of Fe(II)_aq_ concentrations (red dots) and associated isotopic composition (δ^56^Fe_aq_) (black dots) for the ferruginous interval between 200 and 600 mbsf. Error bars indicate the twofold standard deviation (2SD) of the isotope ratio over 20 consecutive measurement cycles. (**b**) Relationship between Fe(II)_aq_ concentrations (expressed as Ln[Fe(II)_aq_]) and δ^56^Fe_aq_ values (data fit following the relation: δ^56^Fe_aq_ = 1.22 * Ln[Fe(II)_aq_] − 6.09, Spearman correlation coefficient r_s_ = 0.77; *p* = 0.0002; n = 18; α = 0.05; two-tailed). The shallowest sample (~ 200 mbsf; gray dot) is not included in the linear regression due co-occurrence of Fe(II)_aq_ and HS^−^ (details given in the text).

The isotopic composition of dissolved Fe (δ^56^Fe_aq_) is < − 1.0‰ over the whole ferruginous (Fe(II)_aq_-enriched) interval between 200 and 600 mbsf (Fig. 2a), thus, lower than the average isotopic composition of igneous rocks (δ^56^Fe = 0.09 ± 0.05‰, 1SD; ref.^17^).

The Fe(II)_aq_ concentrations (expressed as ln[Fe(II)_aq_]) correlate with δ^56^Fe_aq_ values (Spearman correlation coefficient r_s_ = 0.77, *p* = 0.0002, n = 18, α = 0.05, two-tailed; Fig. 2b) except for the shallowest sample at ~ 200 mbsf. The maximum Fe(II)_aq_ concentration of ~ 60 µM at 570 mbsf corresponds to δ^56^Fe_aq_ = − 1.5‰, whereas an extremely low δ^56^Fe_aq_ value of − 5.86‰ at 473 mbsf coincides with a low Fe(II)_aq_ concentration of 2 µM (Fig. 2a). The highest value of − 1.09‰ at ~ 200 mbsf can likely be attributed to reactions between Fe(II)_aq_ and hydrogen sulfide (HS^−^), since local minima of both compounds were detected at the same depth (< 2 µM and < 4 µM, respectively; Supplementary Fig. 2b, c)^25^. Fe sulfide precipitation kinetically favors ^54^Fe over ^56^Fe, leading to higher residual δ^56^Fe_aq_ values^6,22^.

To our knowledge, the δ^56^Fe_aq_ value of − 5.86‰ is the lowest ever measured in marine pore waters. To use Fe isotopes as a proxy for biogeochemical processes, the isotopic composition of the co-occurring solid reactive Fe phases is also required^8,18,23^. Stable Fe isotope analyses were performed on the Fe_aca_ (δ^56^Fe_aca_) and Fe_hyam_ (δ^56^Fe_hyam_) extracts, as variations in the δ^56^Fe values are most likely to occur in these reducible Fe pools^8,18^. The δ^56^Fe_aca_ values of mud rock samples decrease downcore from − 0.33 to − 0.72‰, whereas the δ^56^Fe_aca_ values of ash layers are more variable, ranging between − 0.28 and − 1.05‰ (Fig. 1c). Average δ^56^Fe_hyam_ values are similar for both set of samples with − 0.26 ± 0.09‰ (1SD, n = 9) for mud rock and − 0.27 ± 0.09‰ (1SD, n = 9) for ash samples (Fig. 1d). Given that low ^56^Fe/^54^Fe ratios of Fe(II)_aq_ relative to ferric substrates are often related to DIR with an isotopic fractionation of up to − 3‰ (refs.^6,11,13^), the more negative δ^56^Fe_aq_ values compared to δ^56^Fe_aca_ and δ^56^Fe_hyam_ values first pointed towards DIR. However, the extremely negative δ^56^Fe_aq_ values down to − 5.86‰ cannot be explained by DIR alone. Since equilibrium isotope fractionation generally decreases with increasing temperature proportional to 1/T^2^, the DIR related isotopic fractionation would be even less than − 3‰ (ref.^14^), considering the elevated temperatures of up to ~ 70 °C in the ferruginous zone (Fig. 1a). In the following, we highlight possible reaction pathways that typically cause Fe isotope fractionation in marine sediments and discuss them in terms of their applicability to Site C0023.

The formation of Fe carbonates such as siderite (FeCO_3_) and Ca-substituted siderite, for instance, is associated with a fractionation of up to ~ 1‰ between Fe(II)_aq_ and Fe bound in carbonates whereby the light ^54^Fe is preferentially incorporated into the Fe carbonate precipitate^37^. However, we observe a much greater difference of more than 4‰ between δ^56^Fe_aq_ and δ^56^Fe_aca_ and a relative enrichment of ^54^Fe in the residual Fe(II)_aq_ pool. Similarly, kinetically controlled Fe sulfide precipitation, which we assume to dominate over equilibrium fractionation in natural systems, would also result in higher δ^56^Fe_aq_ values^6,22^. In case of a transition to a dominance of equilibrium fractionation, the fractionation would result in lower δ^56^Fe_aq_ values^38^. Even if an equilibrium has been established over time, the equilibrium fractionation factor of − 0.33 to − 0.52‰ between Fe(II)_aq_ and the Fe sulfide mineral mackinawite^38^, a precursor of pyrite, is most likely insufficient to produce δ^56^Fe_aq_ values of almost − 6‰. Thus, we rule out the precipitation of authigenic Fe sulfide and carbonate minerals as the dominant reaction pathways explaining the observed extremely low δ^56^Fe_aq_ values.

One conceivable scenario leading to extremely negative δ^56^Fe_aq_ values is the repetitive redox cycling of Fe at the Fe(II)/Fe(III) redox boundary. The zone with elevated Fe(II)_aq_ concentrations corresponds to an age of ~ 0.3 to 2.5 Ma (Fig. 1a)^39^. The onset of DIR and, thus, the establishment of the Fe(II)/Fe(III) redox boundary was at ~ 2.5 Ma, when the rate of organic carbon burial increased due to higher sedimentation rates and elevated primary productivity^31^. Numerous ash layers were also deposited during this time period (Fig. 1a), suggesting that volcanic ash could have provided reactive Fe(III) available to Fe-reducing organisms and thus stimulated high rates of DIR.

DIR leads to a preferential release of isotopically light ^54^Fe into the pore water, and consequently negative δ^56^Fe_aq_ values^5,15,16^. As Fe(II)_aq_ diffused up towards the Fe(II)/Fe(III) redox boundary, Fe(II)_aq_ could have been oxidized to Fe(III)_aq_ and precipitated as secondary Fe(III) (oxyhydr)oxides. The oxidation of Fe(II)_aq_ and the subsequent precipitation as solid-phase Fe(III) also results in isotope fractionation^14,20,40^, whereby secondary Fe(III) (oxyhydr)oxides are isotopically heavier than Fe(II)_aq_, but lighter compared to the primary ferric substrate. With ongoing deposition and changes in geochemical conditions, the Fe(II)/Fe(III) redox boundary at Site C0023 presumably migrated upwards^31^ so that secondary Fe(III) (oxyhydr)oxides could have been successively buried and used as energy substrates. The repetitive Fe-reduction–oxidation cycling could have led to a continuous shift of pore-water δ^56^Fe towards more negative values over time. However, a recent study has shown that the population of vegetative cells at Site C0023 sharply drops by two orders of magnitude at 300–400 mbsf, which corresponds to the temperature limit for growth of mesophilic microorganisms of ~ 45 °C, and remains close to the minimum quantification limit further below. The collapse likely occurred when the temperature considerably increased since the onset of trench-style deposition at ~ 0.4 Ma^24^. Therefore, considering the sediment age of 0.3–2.5 Ma^39^, it is unlikely that the present-day isotopic composition of Fe(II)_aq_ still records the repetitive Fe redox cycling that occurred several hundreds of thousands of years ago.

The microbial reduction of Fe(III)-containing clays can be considered as an alternative explanation for the release of Fe(II)_aq_ and its extremely negative isotopic composition^41,42^. Structural Fe(III) in clay minerals can serve as electron acceptor for the degradation of organic matter^43,44^. Recently, Kim et al.^45^ postulated that microbial reduction of structural Fe(III) in smectite promotes the transformation of smectite to illite at Site C0023 between 500 and 700 mbsf, leading to the observed Fe(II)_aq_ release (Fig. 1a). The fractionation between Fe(II)_aq_ and structural Fe(III) in nontronite, an Fe-rich member of the smectite group, ranges between − 1.2 to + 0.8‰ (ref.^41^). If microbial reduction of Fe(III)-containing clays is the only process leading to isotope fractionation, δ^56^Fe_hyam_ would need to be at least − 2.0‰ for samples with δ^56^Fe_aq_ < − 3.5‰. Since we observe much greater differences between δ^56^Fe_aq_ and δ^56^Fe_hyam_ values in all samples, we infer that additional processes are responsible for the uniquely low δ^56^Fe_aq_ values at Site C0023.

### Adsorption of Fe(II)_aq_ onto mineral surfaces

The co-variation between Fe(II)_aq_ concentrations and δ^56^Fe_aq_ (Fig. 2b) could suggest progressive removal of Fe(II)_aq_ in a Rayleigh distillation process^46^. A conceivable scenario in which Fe(II)_aq_ is continuously removed from the pore water is adsorption onto Fe (oxyhydr)oxide surfaces during the diffusional transport of Fe(II)_aq_ along a concentration gradient. In natural aqueous systems, diffusion itself likely only plays a negligible role in controlling Fe isotope fractionation since Fe ions do not diffuse as free ions, but are surrounded by a large solvation shell^47^. The preferential adsorption of isotopically heavy ^56^Fe onto mineral surfaces^15,16,19^ could lead to very low residual δ^56^Fe_aq_, as observed in this study. Fractionation factors between Fe(II)_sorb_ on goethite and Fe(II)_aq_ (Δ^56^Fe_Fe(II)sorb-Fe(II)aq_) are between + 0.87 and + 1.24‰ (refs.^15,16,48^). Since we cannot differentiate between carbonate-bound Fe and Fe(II)_sorb_ within the Fe_aca_ pool, an assessment of the isotopic composition of Fe(II)_sorb_ is not feasible in the framework of this study.

To assess the plausibility of the proposed scenario, we calculated the evolution of the isotopic composition of Fe(II)_aq_ and Fe(II)_sorb_ using the Rayleigh distillation equations after Wiederhold^49^. The isotopic composition of Fe(II)_aq_ is described in good approximation by:

1 $$\delta^{56} Fe_{aq} = \delta^{56} Fe_{0} + \varepsilon \ln f,$$

where δ^56^Fe_0_ is the initial isotopic composition of Fe(II)_aq_, ε is the isotopic enrichment factor in permil and f is the remaining fraction of Fe(II)_aq_. The isotopic composition of the instantaneous product Fe(II)_sorb_ (δ^56^Fe_sorb-instant_) is calculated as:

2 $$\delta^{56} Fe_{sorb - instant} = \delta^{56} Fe_{0} + \varepsilon \ln f + \varepsilon ,$$

and the cumulative product Fe(II)_sorb_ (δ^56^Fe_sorb-cumulative_) as:

3 $$\delta^{56} Fe_{sorb - cumulative} = \, \delta^{56} Fe0 \, + \, \varepsilon \ln f - \frac{\varepsilon \ln f}{{1 - f}}.$$

**Figure 3:**
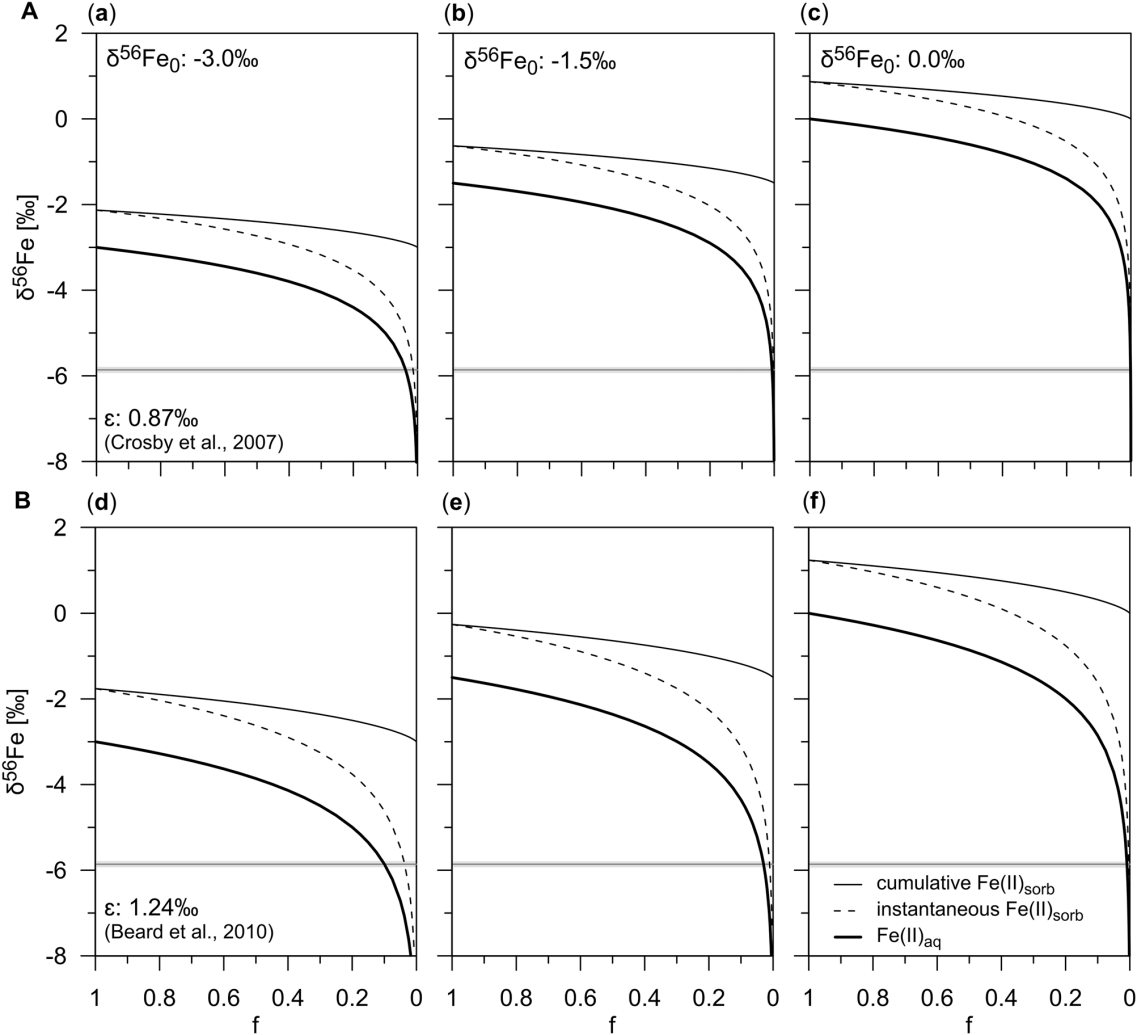
Isotopic composition of dissolved and adsorbed Fe derived from the Rayleigh distillation model. (**a**)–(**f**) Isotopic evolution of Fe(II)_aq_ (bold line), instantaneous Fe(II)_sorb_ (dashed line) and cumulative Fe(II)_sorb_ (narrow line) during the adsorption of Fe(II)_aq_ onto Fe (oxyhydr)oxide surfaces obtained by using the Rayleigh distillation equations after Wiederhold^49^. The isotopic compositions are plotted against f, which is the remaining fraction of Fe(II)_aq_. The differences between Fe(II)_aq_ and instantaneous Fe(II)_sorb_ corresponds to the enrichment factors ε according to (**A**) Crosby et al.^16^ (2007) and (**B**) Beard et al.^48^, respectively, at all stages of the reaction. For the initial isotopic composition of Fe(II)_aq_ (δ^56^Fe_0_), different values of − 3.0‰ (**a**, **d**), − 1.5‰ (**b**, **e**), and 0.0‰ (**c**, **f**) were used. The grayish bar represents the reference δ^56^Fe_aq_ value of − 5.86‰. The model results show that the largest fractionation for Fe(II)_aq_ are found at advanced stages of the adsorption process where the remaining fraction f is low (f < 0.1).

We tested different values for the initial isotopic composition of Fe(II)_aq_ (Fig. 3). We argue that an initial δ^56^Fe_0_ value between − 1.5 and − 3.0‰ is reasonable due to the onset of DIR at ~ 2.5 Ma. For the enrichment factor ε, we have used 0.87‰ (ref.^16^) and 1.24‰ (ref.^48^). It should be noted that the enrichment factors are estimated based on laboratory studies that were performed at room temperature. Fractionation factors in natural systems could be different due to lower or higher in situ temperatures such as at Site C0023 and the availability of competing adsorbents including dissolved silica^50^.

The Rayleigh distillation model results show that extremely low δ^56^Fe_aq_ values of almost − 5.9‰ are reached in all scenarios at advanced stages of the adsorption process when the remaining fraction is low (f < 0.1; Fig. 3). Except for the scenarios with the lowest (f = 0.001; Fig. 3c) and highest (f = 0.1; Fig. 3d) values for f, this corresponds to an initial Fe(II)_aq_ concentration between 55 and 320 µM if considering the actual Fe(II)_aq_ concentration of ~ 2 µM in the sample with the uniquely low δ^56^Fe_aq_ value (Fig. 2b). This calculated range of initial Fe(II)_aq_ concentrations is in line with concentrations typically associated with DIR in shallow sediments^6,18,51,52^. Desorption of Fe(II)_sorb_ cannot be completely excluded. However, if an equilibrium between adsorption and desorption has been established, the fractionation factor would have to be very large to reach extremely low δ^56^Fe_aq_ values, which is rather unrealistic.

The described scenario including DIR-mediated release of Fe(II)_aq_ followed by its adsorptive removal during diffusion only holds true if DIR once occurred but has ceased at that specific depth during progressive burial and heating. If DIR had continued, the successively released Fe(II)_aq_ would have continuously overprinted the isotopic composition of Fe(II)_aq_ by shifting δ^56^Fe_aq_ values towards 0‰. Based on the present-day extremely low vegetative cell population^24^ including Fe-reducing bacteria, we suggest that DIR is currently not occurring or only at very low rates since ~ 0.4 Ma. At the same time, potential reactants such as HS^−^ are not present in pore water in the specific depth interval (Supplementary Fig. 2c). Consequently, Rayleigh distillation—in this case adsorption of Fe(II)_aq_ onto Fe oxide surfaces—could proceed unimpeded over several thousands of years. The proposed Rayleigh distillation model is plausible and we consider it to be the main reason for the extremely low δ^56^Fe_aq_ values at Site C0023. However, it needs to be noted that this site underwent complex diagenetic overprint for millions of years and resolving all past and present Fe fractionating processes, based on the ‘snapshot’ we got from sampling, is impossible.

### Implications for the interpretation of stable Fe isotope data

**Figure 4:**
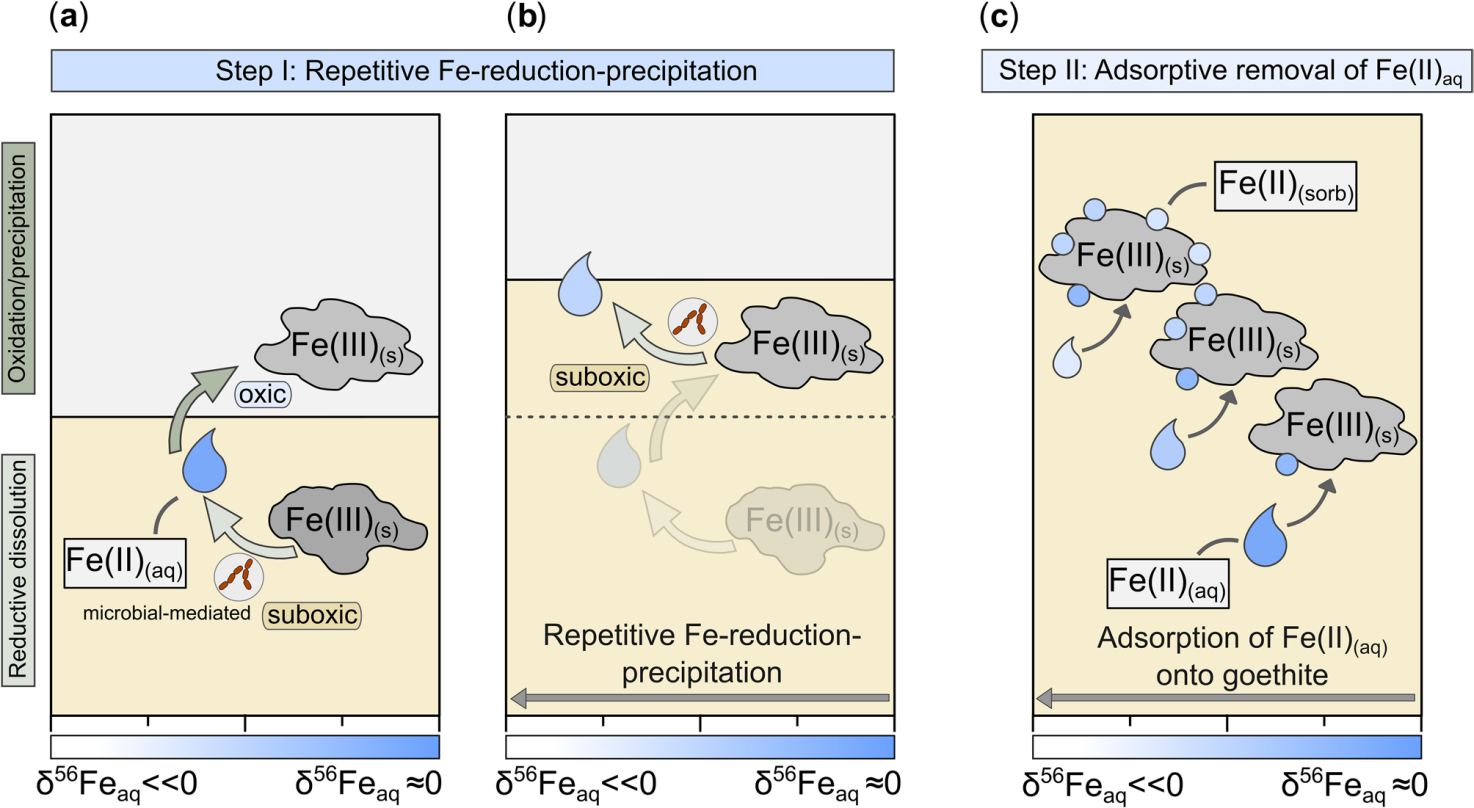
Schematic overview illustrating the shift of δ^56^Fe_aq_ towards negative values. A combination of two consecutive processes could have caused extremely negative δ^56^Fe_aq_ values at Site C0023: (**a**–**b**) repetitive DIR and (**c**) adsorption of Fe(II)_aq_ onto Fe (oxyhydr)oxide surfaces. The blue color scheme represents the isotopic composition of Fe(II)_aq_, where pale blue colors indicate low and dark blue colors higher δ^56^Fe_aq_ values. The combination of repetitive DIR (Step I) and the subsequent adsorption of Fe(II)_aq_ onto mineral surfaces progressively removed Fe(II)_aq_ from the pore water (Step II), which led to a shift of δ^56^Fe_aq_ towards extremely negative values. However, the adsorptive removal of Fe(II)_aq_ (Step II) can only lead to extremely low δ^56^Fe_aq_ values if DIR has ceased (details given in the text). Here, while the zone in which dissimilatory Fe reduction occurs is referred to as ‘suboxic’ zone, where oxygen, nitrate and HS^−^ are absent, the zone in which Fe(II)_aq_ is oxidized is described as ‘oxic’ zone.

This is the first study that reports stable isotopic records of Fe(II)_aq_ in deep subseafloor sediments. We conclude that the detected Fe(II)_aq_ at Site C0023 is vestigial Fe from ancient microbial Fe reduction—possibly of reactive Fe(III) in the ash layers—that has distilled and fractionated over millions of years. Based on the processes outlined, we developed a schematic conceptual model (Fig. 4). Repetitive Fe-reduction–oxidation cycles could have led to increasing negative δ^56^Fe_aq_ values (Fig. 4a, b). We argue that Fe(II)_aq_ release by DIR and adsorptive removal of Fe(II)_aq_ likely co-occurred during this stage. The increase in temperature beyond the temperature limit of mesophilic microorganisms and the associated collapse of the vegetative cell population since the onset of trench-style deposition at ~ 0.4 Ma might have stopped the microbially mediated Fe(II)_aq_ release while the adsorptive Fe(II)_aq_ removal continued (Fig. 4c). Our findings demonstrate that the overall low isotopic composition of Fe(II)_aq_ throughout the ferruginous sediment interval does not rule out microbial reduction as the main pathway releasing Fe(II)_aq_. However, the uniquely low δ^56^Fe_aq_ values are caused by the decoupling of biotic and abiotic processes, which is ultimately driven by the depositional and thermal history of Site C0023. In contrast to deep and consolidated sediments, Rayleigh fractionation probably only plays a minor role in soft near-surface sediments where generally higher reaction rates prevail. This study advances our knowledge about Fe cycling pathways in deep subseafloor sediments and provides crucial aspects for the interpretation of Fe isotope data especially in deep subseafloor sediments. The described process of adsorptive removal of Fe(II)_aq_ and the associated fractionation predominantly influences the dissolved Fe pool. The extremely negative δ^56^Fe_aq_ values occur in the samples with low Fe(II)_aq_ concentrations, which only represent a small proportion of the total Fe content. Hence, the distillation likely does not leave a significant imprint on the Fe isotope record in the solid phase, but only in dissolved Fe pool.

We conclude that the described decoupling of biotic and abiotic processes is important to consider in subseafloor environments where DIR cannot be maintained. Possible reasons for a cessation of DIR can be either the absence of reactive Fe phases as energy substrates as they have been already consumed or changing environmental and depositional conditions—in particular temperature increase beyond the threshold of mesophilic Fe-reducing microorganisms—such as shown for Site C0023.

## Methods

### Pore-water and sediment sampling

Pore-water and solid-phase samples were obtained from whole-round core (WRC) samples onboard *D/V Chikyu* as described in the Method Chapter of the Expedition Report^53^ and by Heuer et al.^24^. Pore water was extracted by squeezing WRC samples in titanium squeezers modified after the stainless steel squeezer of Manheim and Sayles^54^, whereby contact of WRCs and pore water with oxygen was avoided until redox-sensitive parameters have been measured.

For shore-based δ^56^Fe analyses, pore-water aliquots were acidified (100 µl/1 ml sample volume) with concentrated ultrapure HCl (TAMAPURE AA-100 grade, Tama Chemicals Co. Ltd., Japan) directly after sampling and stored in pre-cleaned vials at + 4 °C. The remaining solid-phase samples (i.e., whole-round squeeze cakes) were transferred into gas-tight aluminum bags, flushed with nitrogen, vacuum-sealed, and stored at − 20 °C for further solid-phase analyses. In addition to whole-round squeeze cakes, solid-phase samples taken from discrete ash layers were analyzed in the framework of this study. The discrete ash layers were visually identified and samples were obtained from the working halves (Supplementary Fig. 3). All sediment depths in this study are given as corrected core composite depths below seafloor (CCSF-B) in meters below seafloor (mbsf).

### Pore-water analyses

Pore-water constituents were analyzed onboard *D/V Chikyu* and are described in detail in the Method Chapter of the Expedition Report^53^ and in Köster et al.^31^. Briefly, dissolved Fe (Fe(II)_aq_) was determined using the ferrozine method after Stookey^55^.

Pore-water samples for δ^56^Fe analyses (n = 19) were processed in the laboratory at the Alfred Wegener Institute (AWI) Helmholtz Centre for Polar and Marine Research in Bremerhaven, Germany. After evaporation and re-dissolution in 5M HCl + 0.001% v/v suprapur® H_2_O_2_, pore-water Fe was purified from sample matrices by column chromatography using the AG-MP1 anion exchange resin according to Homoky et al.^56^. The Fe eluate was dried and re-dissolved in 1 ml of 0.3M HNO_3_. HCl and HNO_3_ were of sub-boiling distilled quality. In order to exclude Fe loss during column separation, sample loading and matrix elements eluting fractions were collected in separate vials and aliquots of each sample were analyzed by inductively coupled plasma-mass spectrometer (ICP-MS; Element 2, Thermo Fisher Scientific Inc.). The loss of Fe was < 2% of the total Fe concentration in all samples. Column calibrations with artificial pore-water samples confirmed the effective extraction of Fe from the salt matrix (e.g., Na, Ca, Mg, S) and trace metals such as Ni and Cr (Supplementary Fig. 5).

The Fe isotope measurement was performed using a Multicollector-inductively coupled plasma-mass spectrometer (MC-ICP-MS) (ThermoFinnigan Neptune) at the University of Cologne, Germany. The purified samples were measured by ICP-MS (Element 2, Thermo Fisher Scientific Inc.) and sub-samples of 0.2 ppm (± 10%) were prepared for MC-ICP-MS analysis (Fe matching). The Neptune was equipped with an ESI Apex-Q desolvating system and standard (H) nickel cones. We used the sample-standard-bracketing (SSB) approach with the isotopic reference material (RM) IRMM-524 to correct instrumental mass bias^57^. Data are reported as:

$$\delta^{{{56}}} {\text{Fe }}\left[ \textperthousand \right]{ = }\left[ {\left( {^{{{56}}} {\text{Fe}}/^{{{54}}} {\text{Fe}}_{{{\text{sample}}}} } \right)/\left( {^{{{56}}} {\text{Fe}}/^{{{54}}} {\text{Fe}}_{{{\text{IRMM}} - {524}}} } \right) - {1}} \right] \, *{1}000.$$

The instrumental reproducibility was monitored using the internal laboratory RM JM (Johnson&Matthey, Fe Puratronic wire). The measured δ^56^Fe values for the JM samples (0.42 ± 0.06‰, 2SD, n = 19; Supplementary Fig. 6) were similar to the target value of 0.42 ± 0.05‰ (2SD; ref.^57^). Uncertainty for the individual samples is expressed as twofold standard deviation (2SD) of the isotope ratio over 20 consecutive measurement cycles (Fig. 2b). Duplicate sample measurements (n = 5) were within the uncertainty of the respective individual samples (2SD).

Two RMs of known isotopic composition (a granite rock (AC-E; Ailsa Craig Island, Scotland; Service d’Analyse des Roches et des Minéraux (SARM) and the internal laboratory standard JM; 2 and 4 ppm Fe each) underwent the same chemical processing to verify analytical accuracy and preclude Fe isotope fractionation during column chromatography. The measured δ^56^Fe values were: 1) 0.31 ± 0.06‰ (2SD, n = 8) for the RM AC-E and 2) 0.45 ± 0.09‰ (2SD, n = 4) for the internal laboratory RM JM and, thus, within analytical uncertainty of the target values (AC-E: 0.320 ± 0.010‰, 2SDmean (ref.^58^); JM: 0.42 ± 0.05‰, 2SD (Schoenberg and von Blanckenburg, 2005); Supplementary Fig. 7).

### Solid-phase analyses

Solid-phase analyses include total acid digestions, sequential extraction of different reactive Fe pools and δ^56^Fe analyses of extracted Fe. While bulk Fe, Al and Ti and reactive Fe contents of whole-round squeeze cake samples were taken from Köster et al.^31^, all solid-phase data of ash layer samples and δ^56^Fe analyses were conducted in this study. Total acid digestions and sequential extractions of ash layer samples were performed similarly as described in Köster et al.^31^. All solid-phase analyses were conducted in the laboratory at the AWI.

Total acid digestions were performed using a CEM Mars Xpress microwave system. Bulk element contents of Fe, Mn, Al and Ti were determined by inductively coupled plasma optical emission spectrometry (ICP-OES; iCAP 7400, Thermo Fisher Scientific Inc.) analysis under application of an internal yttrium standard. The RM NIST SRM 2702 and the RM MESS-4 were processed with each set of samples to monitor analytical accuracy. Recoveries were 93.4 ± 2.8% (2SD) for Fe, 93.9 ± 4.0% (2SD) for Mn, 91.3 ± 2.4% (2SD) for Al and 94.7 ± 3.5% (2SD) for Ti for NIST SRM 2702 (n = 4) and 100.7 ± 3.6% (2SD) for Fe, 93.8 ± 5.5% (2SD) for Mn, 90.5 ± 4.6% (2SD) for Al and 95.1 ± 4.2% (2SD) for Ti for MESS-4 (n = 4).

Sequential extractions were performed following the protocols described by Poulton and Canfield^26^ and Henkel et al.^18^ (Supplementary Tab. 1). An internal laboratory RM (HE443-077-cc; anoxic sediment from the Helgoland mud area, North Sea) was processed in each batch of samples to determine the analytical precision. Repetitive analyses of the internal laboratory RM resulted in values similar to the respective Fe and Mn contents determined over the previous years in the laboratories at the AWI (Supplementary Tab. 2).

For δ^56^Fe analyses, the Na-acetate- and hydroxylamine-HCl-leached extracts (Fe_aca_ and Fe_hyam_) were further processed following the protocol by Henkel et al.^18^. After repeated oxidation in a mixture of distilled HNO_3_, HCl and suprapur® H_2_O_2_, Fe precipitation and anion exchange chromatography after Schoenberg and von Blanckenburg^57^, the purified samples were matched to 0.5 ppm (± 10%) following ICP-OES analysis.

Iron isotope measurements of extracted Fe pools were performed on a MC-ICP-MS Neptune plus (ThermoScientific) at MARUM—Center for Marine Environmental Sciences, University of Bremen. The instrument was equipped with a normal interface and the stable introduction system (SIS) including a low flow PFA nebulizer (50 µl) and a cyclonic/Scott quartz spray chamber that was combined with a high efficiency X-cone. Similar to the pore-water δ^56^Fe measurement, we applied SSB with the isotopic RM IRMM-014 and the internal laboratory RM JM was measured to check analytical reproducibility of the analyses. The average δ^56^Fe value of the JM samples was 0.43 ± 0.08‰ (2SD, n = 65; Supplementary Fig. 6) Sub-samples of the Fe standard solution Certipur® to which the extracting reagents Na-acetate and hydroxylamine-HCl were added and the Fe_aca_ and Fe_hyam_ extracts of the internal laboratory RM HE443-077-cc underwent the same purification processing as the samples. Values for the Certipur® standards were 0.12 ± 0.09‰ (2SD) for Na-acetate (n = 2) and 0.17 ± 0.04‰ (2SD) for hydroxylamine-HCl (n = 2) and are, thus, within standard deviation of the unprocessed solution (δ^56^Fe = 0.15 ± 0.06‰, 2SD) as given in Henkel et al.^18^. The δ^56^Fe values for the Fe_aca_ and Fe_hyam_ extracts of the internal laboratory RM HE443-077-cc in this study are within the analytical uncertainty of δ^56^Fe values determined over the past five years (Supplementary Tab. 2). Uncertainty for δ^56^Fe indicate the twofold standard deviation (2SD) of duplicate to fourfold measurements.

## Supplementary Information

Supplementary Information.

## Data Availability

The data generated in this study (stable Fe isotope data of dissolved Fe and sequentially extracted Fe pools as well as solid-phase geochemistry data of the ash layer samples) are archived in the World Data Center PANGAEA via https://doi.pangaea.de/10.1594/PANGAEA.959760^59^. All other data relevant for this study are reported in the Expedition Report (pore-water data)^25^ or archived in the World Data Center PANGAEA via https://doi.org/10.1594/PANGAEA.930858 (solid-phase geochemistry data of mud rock samples)^60^.
