# Supplementary material for: Uniquely low stable iron isotopic signatures in deep marine sediments caused by Rayleigh distillation
Source: Sci Rep. 2023 Jun 24;13:10281. doi: 10.1038/s41598-023-37254-2 (PMC10290645; doi:10.1038/s41598-023-37254-2)
Supplement: Supplementary file 1 — Supplementary Information. [file 41598_2023_37254_MOESM1_ESM.docx]

**Supplementary Information for:**

**Uniquely low stable iron isotopic signatures in deep marine sediments caused by Rayleigh distillation**

Male Köster^1,2*^, Michael Staubwasser^3^, Anette Meixner^2,4^, Simone A. Kasemann^2,4^, Hayley R. Manners^5^, Yuki Morono^6^, Fumio Inagaki^7,8^, Verena B. Heuer^4^, Sabine Kasten^1,2,4^ and Susann Henkel^1^

^1^Alfred Wegener Institute Helmholtz Centre for Polar and Marine Research, Bremerhaven, Germany.

^2^University of Bremen, Faculty of Geosciences, Bremen, Germany.

^3^University of Cologne, Cologne, Germany.

^4^MARUM – Center for Marine Environmental Sciences, University of Bremen, Bremen, Germany.

^5^School of Geography, Earth and Environmental Sciences, University of Plymouth, Plymouth, United Kingdom.

^6^Kochi Institute for Core Sample Research, Extra-cutting-edge Science and Technology Avant-garde Research (X-star), Japan Agency for Marine-Earth Sciences and Technology (JAMSTEC), Nankoku, Kochi, Japan.

^7^Institute for Marine-Earth Exploration and Engineering (MarE3), JAMSTEC, Yokohama, Japan.

^8^Department of Earth Sciences, Graduate School of Science, Tohoku University, Sendai, Japan.

^*^e-mail: male.koester@awi.de

**This file includes:**

Supplementary Figures 1-7

Supplementary Tables 1-2

Supplementary text: Sedimentary setting and geological background

Supplementary References

**Supplementary Figures**


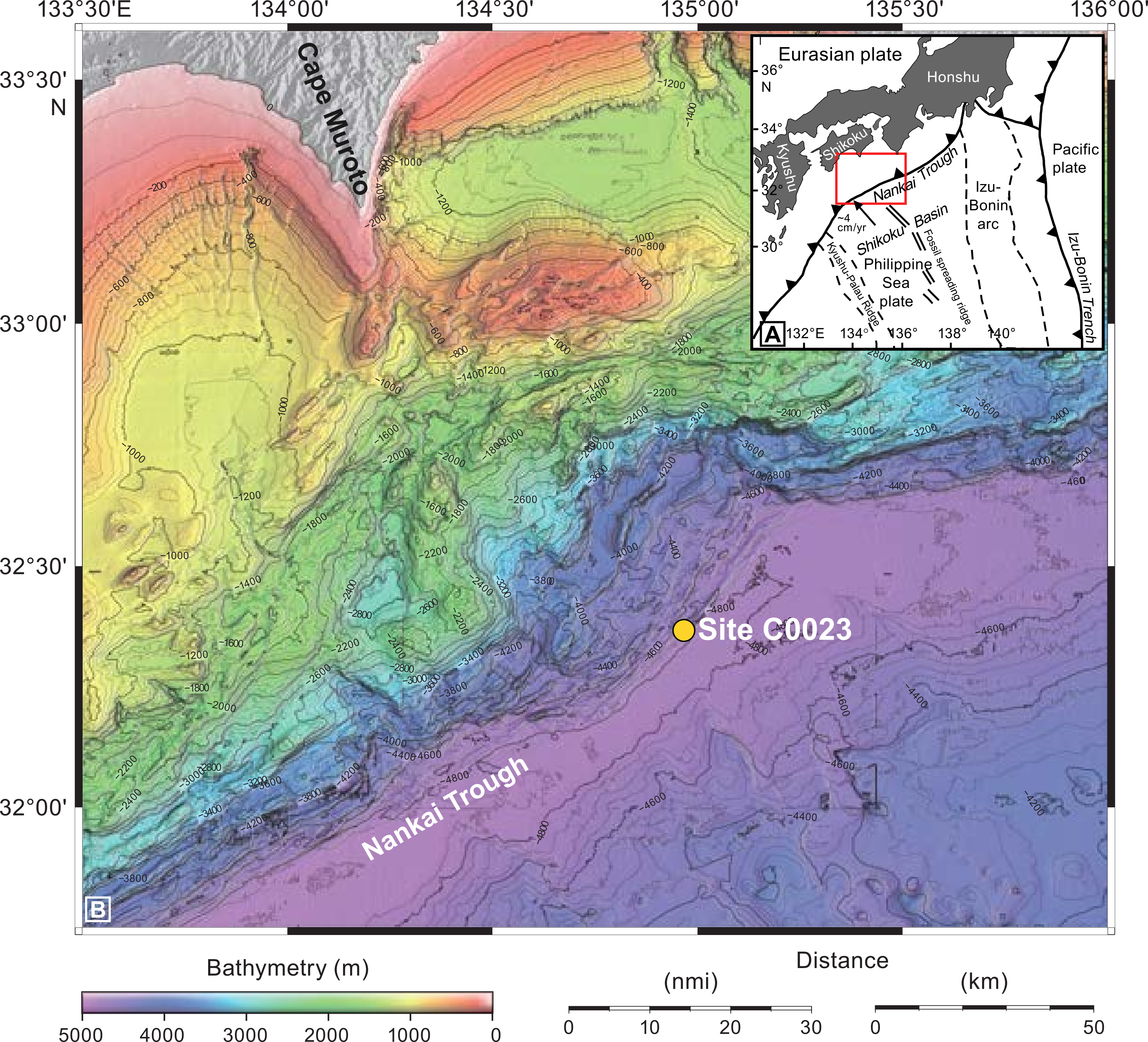


**Figure S1.** Location of IODP Site C0023 in the Nankai Trough offshore Japan. (**A**) Inset showing the general tectonic setting of the Nankai Trough, which marks the subduction boundary between the Philippine Sea plate and the Eurasian Plate. (**B**) Bathymetric map of the Nankai Trough off Cape Muroto. IODP Site C0023 is located in a water depth of 4776 m. Modified after Heuer et al. (2017) (https://creativecommons.org/licenses/by-nc/4.0/). The map was created using the Free and Open Source QGIS (Version 2.0; http://www.qgis.org).


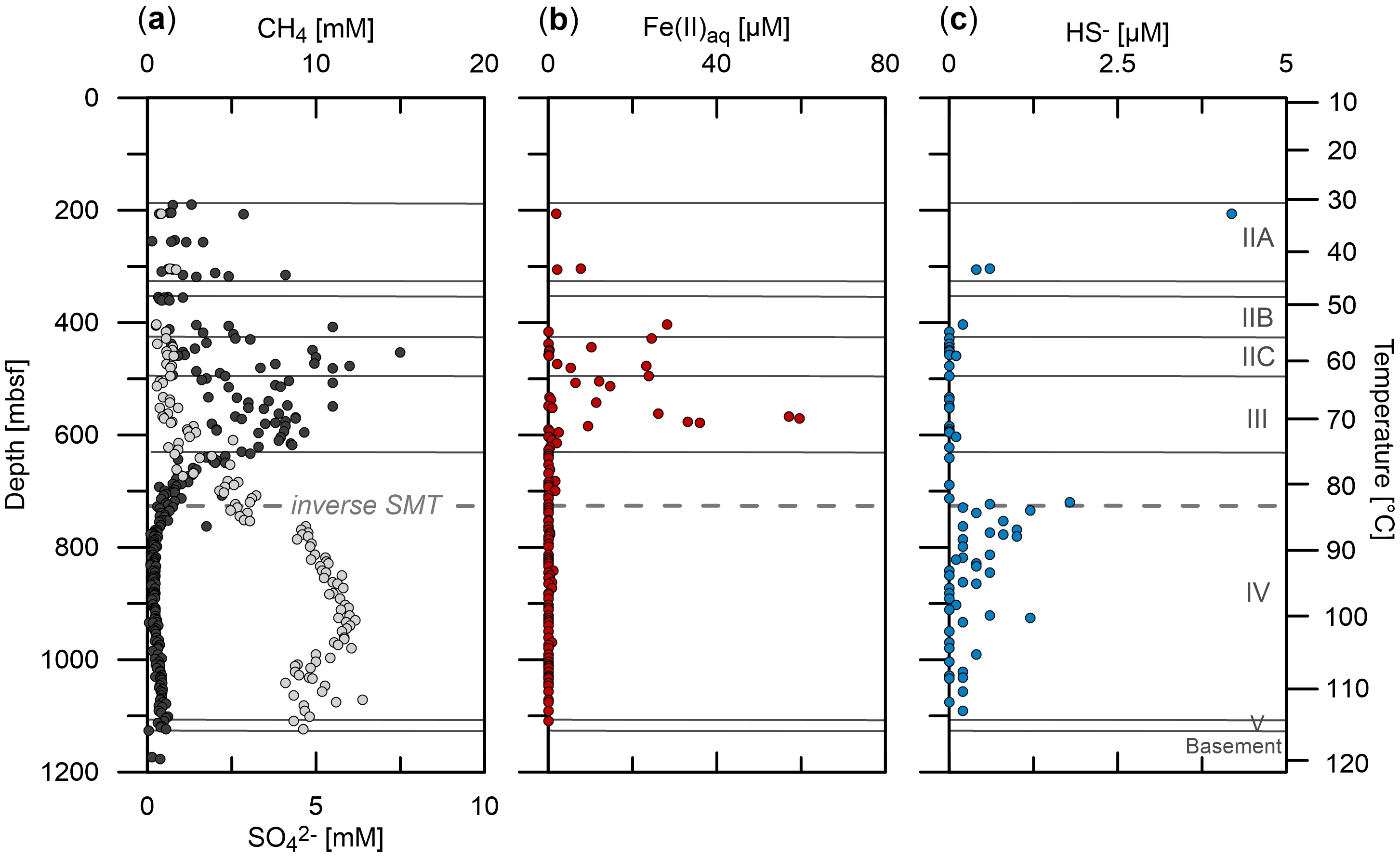


**Figure S2.** Down-core profiles of methane, sulfate, dissolved iron and hydrogen sulfide at Site C0023. Pore-water and gas concentrations of (**a**) methane (CH_4_) (black dots) and sulfate (SO_4_^2-^) (gray dots), (**b**) dissolved iron (Fe(II)_aq_) and (**c**) hydrogen sulfide (HS^-^) (from Heuer et al., 2017). Lithological units (see Sedimentary setting and geological background) and temperature data are from Heuer et al. (2017) and Heuer et al. (2020), respectively. An inverse sulfate-methane transition (SMT) is located at ~730 mbsf.


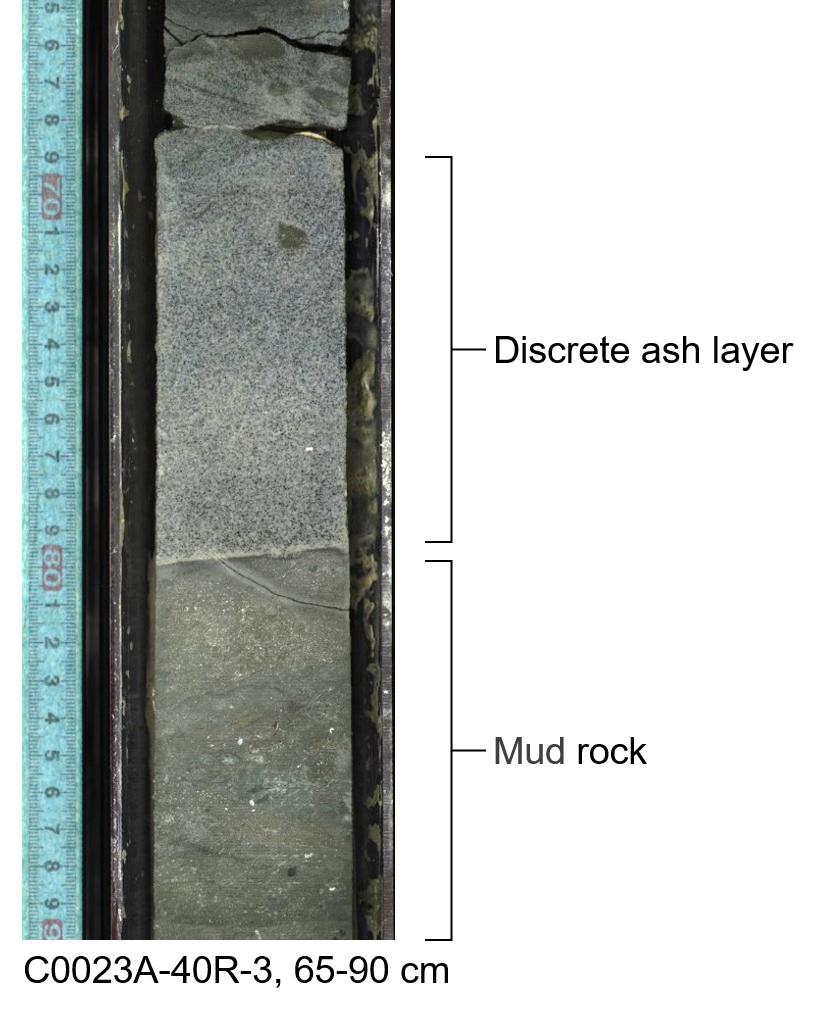


**Figure S3.** Core picture showing a discrete ash layer. The discrete ash layer in core C0023A-40R-3 is located in a depth of ~624 mbsf (from Heuer et al., 2017).


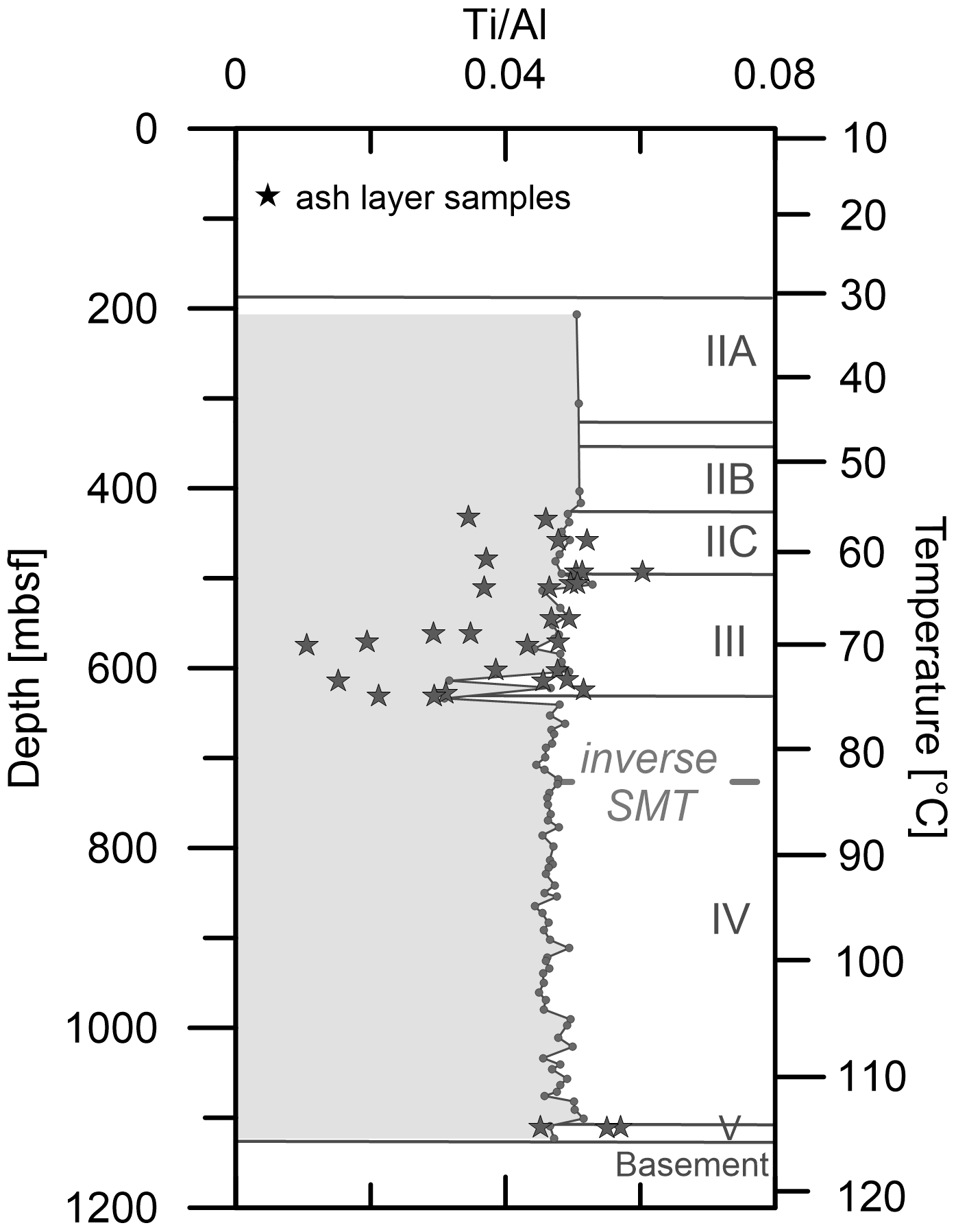


**Figure S4.** Down-core profile of the Ti/Al ratio in mud rock and discrete ash layers at Site C0023. While the Ti/Al ratios in the mud rock samples are relatively constant around 0.05 (gray shaded area), the Ti/Al ratios in the discrete ash layer samples (stars) vary between 0.01 and 0.06. Lithological units and temperature data as in Fig. S2.


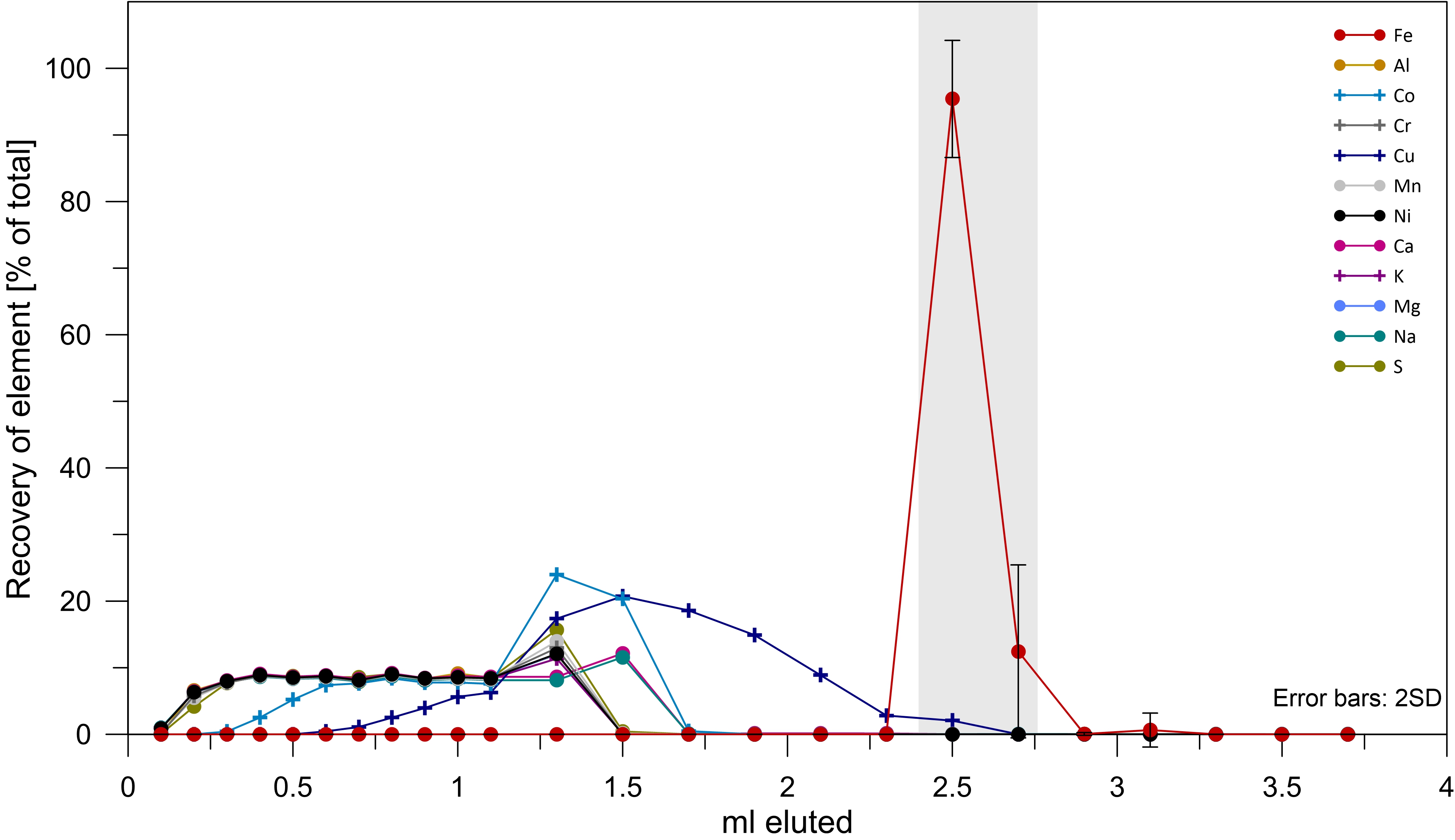


**Figure S5.** Column calibration using the AG-MP1 anion exchange resin. Column calibration confirmed the effective separation of Fe from the salt matrix (Na, K, Ca, Mg, S) and other trace metals (e.g., Ni, Cr, Mn, Co, Cu) by using the AG-MP1 anion exchange resin according to Homoky et al. (2013). The recovery of total Fe is 108% and can be attributed to a dilution effect before ICP-OES measurement. Error bars indicate the twofold standard deviation (2SD; n=4).


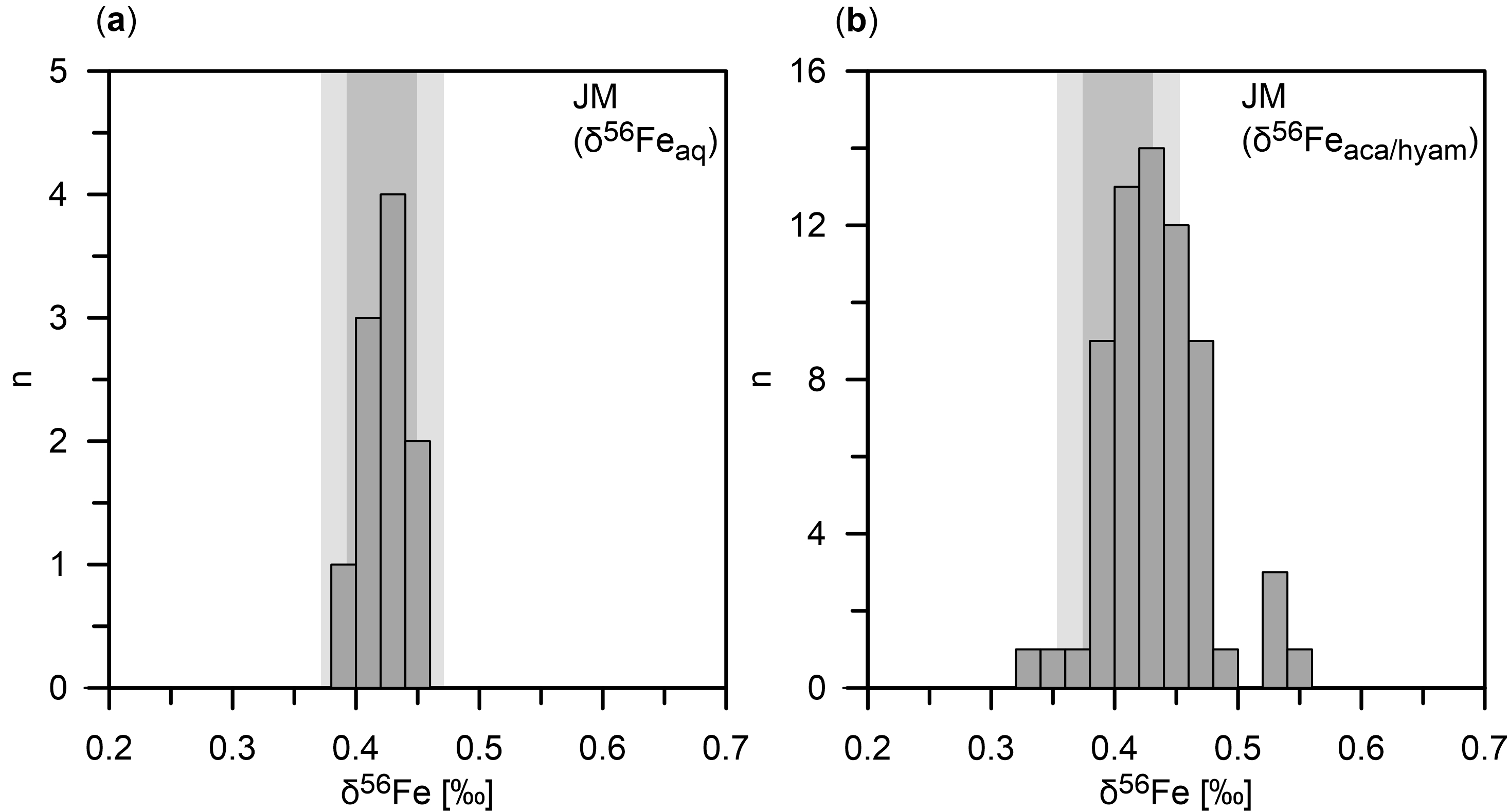


**Figure S6.** JM standard distribution. The internal laboratory RM JM (Johnson&Matthey, Fe puratronic wire) was measured every six samples to monitor the instrumental reproducibility. Only samples in between the JM samples that were within the analytical uncertainty of the target value (0.42 ± 0.05‰, 2SD; Schoenberg and von Blanckenburg, 2005) were taken into account for further data evaluation. The measured δ^56^Fe values for the RM JM were (**a**) 0.42 ± 0.06‰ (2SD, n=19) for the isotopic measurements of pore-water Fe (δ^56^Fe_aq_) and (**b**) 0.43 ± 0.08‰ (2SD, n=65) for the isotopic measurement of the Na-acetate- and hydroxylamine-HCl-leached Fe pools (δ^56^Fe_aca_ and δ^56^Fe_hyam_). The dark and light grayish area represents 1SD and 2SD of target value of the RM JM (Schoenberg and von Blanckenburg, 2005), respectively.


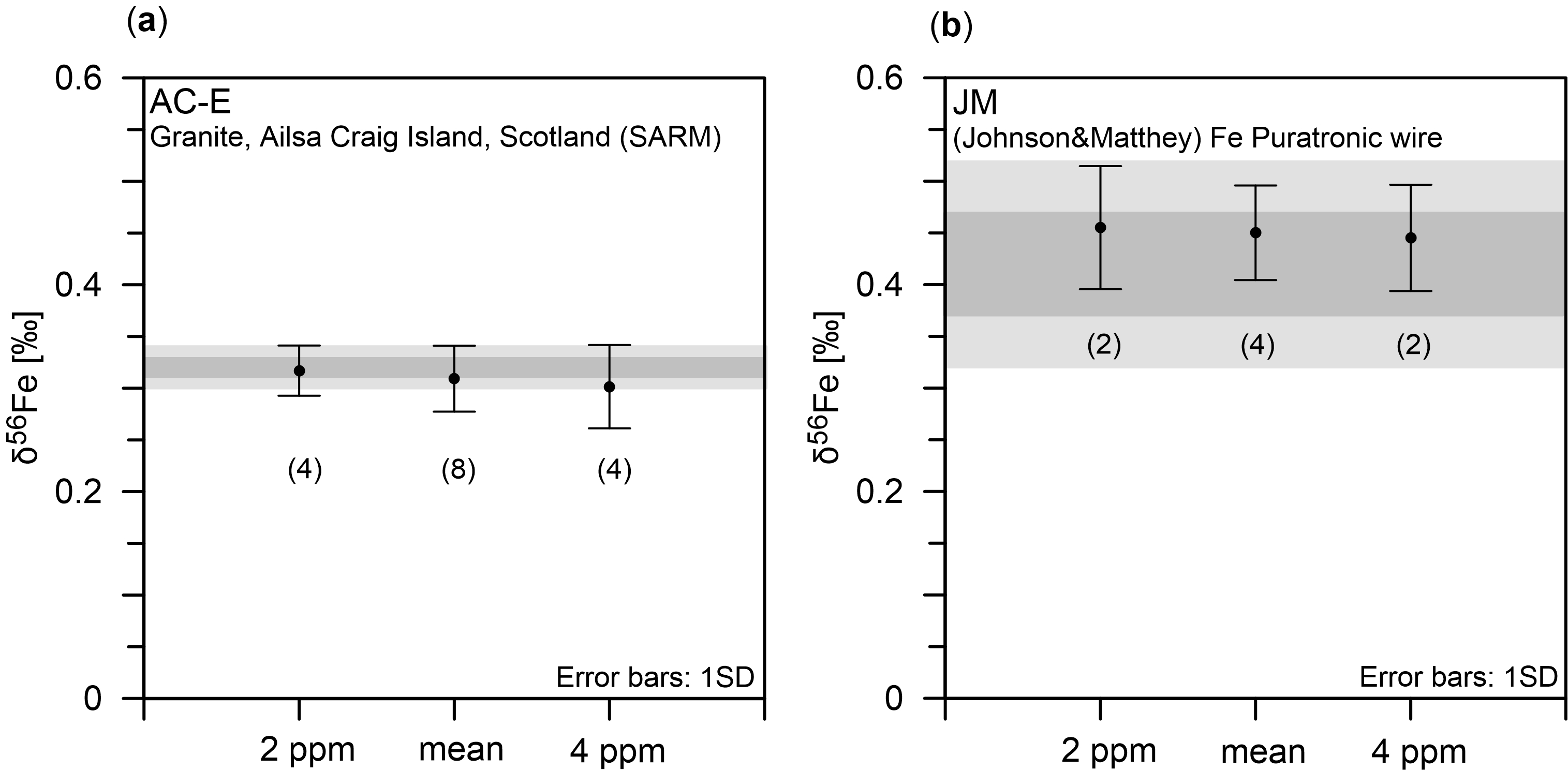


**Figure S7.** Column chromatography of the reference samples AC-E and JM. Isotopic composition of (**a**) the RM AC-E (granite rock; Ailsa Craig Island, Scotland (SARM)) and (**b**) the internal laboratory RM JM (Johnson&Matthey; Fe Puratronic wire) after column chromatography with AG-MP1 anion exchange resin. Error bars indicate the simple standard deviation (1SD). The dark and light grayish area represents 1SD and 2SD of the certified reference values, respectively. Numbers within parentheses refer to the number of processed reference samples for the respective concentration (2 and 4 ppm) and the resulting mean.

**Supplementary Tables**

**Table S1**. Sequential extraction procedure after Poulton and Canfield (2005) applied to mud rock and volcanic ash layer samples of Site C0023.

| **Step** | **Target mineral phase** | **Terminology** | **Extraction reagent** | **Extraction time** | **pH** |
| --- | --- | --- | --- | --- | --- |
| I | Carbonate-bound and sorbed Fe and AVS**^a^** | Fe_aca_ | 1 M Na-acetate | 24 h | 4.5 |
| II | Easily reducible Fe (oxyhydr) oxides (ferrihydrite, lepidocrocite) | Fe_hyam_ | 1 M hydroxylamine-HCl in 25% v/v acetic acid | 48 h |  |
| III | Reducible Fe oxides (goethite, hematite) | Fe_di-ct_ | Na-dithionite (50 g L^-1^)/0.02 M Na-citrate**^b^** | 2 h | 4.8 |
| IV | Magnetite | Fe_oxa_ | 0.2 M ammonium oxalate/0.17 M oxalic acid | 6 h |  |

**^a^**Acid volatile sulfide

**^b^**Na-citrate solution was modified to 0.02 M according to Henkel et al. (2016)

**Table S2**. Sequentially extracted Fe contents in the internal laboratory reference material (RM) HE443-077-cc (anoxic sediment from the Helgoland mud area, North Sea) and associated isotopic composition (δ^56^Fe_aca_ and δ^56^Fe_hyam_) determined over the past 5 years in comparison with Fe contents and isotopic composition determined in the framework of this study. Uncertainty is expressed as the twofold standard deviation (2SD).

| **Step** | **Terminology** | **Fe contents determined over the past 5 years [mg/g] (n=67)** | **Fe contents this study [mg/g] (n=6)** | **δ^56^Fe determined over the past 5 years [‰] (n=4)** | **δ^56^Fe this study [‰] (n=2)** |
| --- | --- | --- | --- | --- | --- |
| I | Fe_aca_ | 4.7 ± 0.6 | 4.8 ± 0.1 | -0.07 ± 0.04 | -0.06 ± 0.03 |
| II | Fe_hyam_ | 3.1 ± 0.7 | 2.8 ± 0.2 | -0.33 ± 0.07 | -0.28 ± 0.04 |
| III | Fe_di-ct_ | 2.6 ± 0.4 | 2.6 ± 0.1 | - | - |
| IV | Fe_oxa_ | 1.0 ± 0.2 | 1.2 ± 0.05 | - | - |

**Sedimentary setting and geological background**

Pore-water and solid-phase samples analyzed in this study were taken from Hole C0023A (Supplementary Fig. 1) during International Ocean Discovery Program (IODP) Expedition 370 (Temperature Limit of the Deep Biosphere off Muroto) onboard *D/V Chikyu* in September-November 2016 (Heuer et al., 2017). IODP Site C0023 (32°22.00´N, 134°57.98´E; 4776 m water depth) is located in the Nankai Trough off Shikoku Island, Japan, where the Philippine Sea plate is subducting beneath the Eurasian plate at a current rate of 4-6 cm yr^-1^ (Seno et al., 1993; DeMets et al., 2010). Due to off-scraping of sediment from the descending Philippine Sea plate, the Nankai accretionary prism has been formed (Le Pichon et al., 1987; Taira et al., 1992). Site C0023 is situated in the protothrust zone of the accretionary prism, where a ~1200 m thick sediment sequence accumulated on the ~16-million-year-old basaltic basement. The sediment sequence at Site C0023 is divided into the following lithostratigraphic units from bottom to top: the volcaniclastic facies (Unit V), the Lower Shikoku Basin facies (hemipelagic mudstone; Unit IV), the Upper Shikoku Basin facies (hemipelagic mudstone with abundant volcanic ash layers; Unit III), the basin-to-trench transitional facies (Subunit IIC), the outer trench-wedge facies (Subunit IIB), and the axial trench-wedge facies (Subunit IIA) (Heuer et al., 2017). A more detailed description including the diagenetic history of Site C0023 can be found in Köster et al. (2021).

**References**

Berner, R. A. Early Diagenesis: A Theoretical Approach (Princeton Univ. Press, Princeton, New Jersey, 1980)

Boudreau, B.P. *Diagenetic Models and Their Implementation: Modelling Transport and Reactions in Aquatic Sediments* (Springer, Berlin, Heidelberg, New York, 1997).

DeMets, C., Gordon, R. G. & Argus, D. F. Geologically current plate motions. *Geophys, J. Int*. **181**, 1-80. https://doi.org/10.1111/j.1365-246X.2009.04491.x (2010).

El-Dessouky, H. T. & Ettouney, H.M. *Fundamentals of Salt Water Desalination* (Elsevier Science B.V., Amsterdam, 2002).

Heuer, V. B. et al. Temperature limit of the deep biosphere off Muroto. *Proceedings of the International Ocean Discovery Program* (Vol. 370). College Station, TX: International Ocean Discovery Program. https://doi.org/10.14379/iodp.proc.370.2017 (2017).

Heuer, V. B. et al. Temperature limits to deep subseafloor life in the Nankai Trough subduction zone. *Science* **370**, 1230-1234. https://doi.org/10.1126/science.abd7934 (2020).

Homoky, W. B., John, S. G., Conway, T. M. & Mills, R. A. Distinct iron isotopic signatures and supply from marine dissolution. *Nat. Commun*. **4**, 1-10. https://doi.org/10.1038/ncomms3143 (2013).

Köster, M. et al. Evolution of (bio-)geochemical processes and diagenetic alteration of sediments along the tectonic migration of ocean floor in the Shikoku Basin off Japan. *Geochem. Geophys. Geosyst*. **22**, e2020GC009585. https://doi.org/10.1029/2020GC009585 (2021).

Le Pichon, X. et al. Nankai Trough and Zenisu Ridge: a deep-sea submersible survey. *Earth Planet. Sci. Lett*. **83**, 285-299. https://doi.org/10.1016/0012-821X(87)90072-0 (1987).

Li, Y.-H., & Gregory, S. Diffusion of ions in sea water and in deep-sea sediments. *Geochim. Cosmochim. Acta* **38**, 703-714. https://doi.org/10.1016/0016-7037(74)90145-8 (1979).

Poulton, S.W. & Canfield, D.E. Development of a sequential extraction procedure for iron: implications for iron in partitioning in continentally derived particulates. *Chem. Geol*. **214**, 209-221. https://doi.org/10.1016/j.chemgeo.2004.09.003 (2005).

Schoenberg, R. & von Blanckenburg, F. An assessment of the accuracy of stable Fe isotope ratio measurements on samples with organic and inorganic matrices by high-resolution multicollector ICP-MS. *Int. J. Mass Spectrom*. **242**, 257-272. https://doi.org/10.1016/j.ijms.2004.11.025 (2005).

Seno, T., Stein, S. & Gripp, A. E. A model for the notion of the Philippine Sea Plate consistent with NUVEL-1 and geological data. *J. Geophys. Res.: Solid Earth* **98**, 17941-17948. https://doi.org/10.1029/93JB00782 (1993).

Taira, A. et al. Sediment deformation and hydrogeology of the Nankai Trough accretionary prism: Synthesis of shipboard results of ODP Leg 131. *Earth Planet. Sci. Lett*. **109**, 431-450. https://doi.org/10.1016/0012-821X(92)90104-4 (1992).
